# Geographic and Socioeconomic Determinants of Full Coverage COVID-19 Vaccination in Peru: Findings from a National Population-Based Study

**DOI:** 10.3390/vaccines11071195

**Published:** 2023-07-03

**Authors:** Akram Hernández-Vásquez, Rodrigo Vargas-Fernández, Carlos Rojas-Roque

**Affiliations:** 1 Centro de Excelencia en Investigaciones Económicas y Sociales en Salud, Vicerrectorado de Investigación, Universidad San Ignacio de Loyola, Lima 15024, Peru; 2 Facultad de Ciencias de la Salud, Universidad Científica del Sur, Lima 15067, Peru; 3 Centre for Health Economics, University of York, York YO10 5DD, UK

**Keywords:** COVID-19, vaccination, COVID-19 vaccines, vaccination coverage, socioeconomic factors, adult, Peru

## Abstract

Despite the fact that vaccination coverage against COVID-19 has made great progress in Peru, there is still a quarter of the population that has not been fully vaccinated. This study aims to determine the factors associated with complete vaccination in Peruvian adults. An analysis of the National Household Survey 2022 in Peru was performed. Prevalence ratios with their 95% confidence intervals (95% CI) were estimated to assess the factors associated with vaccination with three or more doses of the COVID-19 vaccine. A total of 58,471 participants were included in the study and 75.8% of the surveyed population were found to have received full vaccination. Significant differences in complete coverage were observed according to sex, age, educational level, ethnicity, poverty status, and geographic location. In the adjusted analysis, individuals aged 60 years or older, those with higher educational attainment, the non-poor, and those living in urban areas were more likely to be fully vaccinated. Native individuals and people who live in households without media are less likely to be fully covered. These results highlight the importance of considering demographic and socioeconomic factors when analyzing COVID-19 vaccination coverage. Additional strategies are needed to address vaccination gaps and ensure better vaccination coverage.

## 1. Introduction

Coronavirus disease (COVID-19) is considered a health crisis that has had an unprecedented economic, health, and social impact worldwide [1]. Due to the rapid spread of the causative agent (SARS-CoV-2 virus) and its high mortality, several preventive strategies were initiated to control the spread and severity of the disease and lessen its impact [2]. One of the most awaited and effective strategies to control disease severity was vaccination against COVID-19 [3]. The World Health Organization (WHO) reported that the COVID-19 vaccination strategy minimized the number of deaths and severe cases and controlled the negative impact on health systems and the resumption of socioeconomic activities [4], as described in the literature [5,6]. By May 2023, it is estimated that more than 13 billion doses of vaccines have been administered worldwide, and more than five billion people have been vaccinated with at least one dose [7]. However, economic, structural, and individual barriers have not allowed complete coverage of the COVID-19 vaccine in the populations of low- and middle-income countries (LMICs) [8].

In LMICs, the initiation of vaccination against COVID-19 was delayed compared to high-income countries, and even the development of this strategy was slow due to late vaccine procurement or high vaccine costs [9]. In Latin America and the Caribbean, it is estimated that by May 2023, more than 1 billion vaccines will have been administered, more than 380 million people will have a complete vaccination schedule, and more than 300 million booster doses will have been administered [10]. The countries with the highest complete vaccination coverage are Puerto Rico (95.1%), Chile (92.2%), Nicaragua (90.9%), and Cuba (88.5%), while the countries with the lowest coverage are Haiti (2.6%), French Guiana (29.5%), and Guadeloupe (36.5%) [10]. In Peru, the vaccination strategy against COVID-19 started in February 2021 due to a late acquisition of vaccines and prioritized health personnel and people aged 60 years or older due to their greater vulnerability and the lethality of the disease [11]. In October 2021, the Peruvian Ministry of Health initiated mandatory vaccination (two doses) for persons aged 18 years or older throughout the national territory [12] and the third dose in November 2021 [13]. According to the Peruvian Ministry of Health, the complete vaccination schedule includes the administration of three doses of the vaccine (the third as a booster dose) [13]. Thus, currently, it is estimated that approximately 75% of the population throughout the country have a complete vaccination schedule. However, 25% of the Peruvian population has not yet achieved full coverage with the COVID-19 vaccine [14].

According to the biomedical literature, there are socioeconomic and geographic factors that limit the administration or acceptance of vaccination against COVID-19 in the population [15]. Among the socioeconomic factors, several studies have reported that being female, younger, with lower educational levels and income, belonging to ethnic minorities, and having a fear of adverse events of the vaccine were associated with greater refusal or lower willingness for vaccination against COVID-19 [15]. In LMIC, geographic barriers and structural limitations, such as an inadequate storage system, inadequate distribution of vaccines in communities located in rural or remote areas, and lack of access to rural or very remote territories, have compromised compliance with the COVID-19 vaccination schedule [8]. In Peru, several studies have determined factors that would decrease the intention to vaccinate against COVID-19, such as female sex, rural area of residence, low educational level, non-health personnel, and low human development index [16,17,18]. However, these data were collected during the initial stages of vaccination through a virtual questionnaire (which limits its accessibility to people who do not have access to the Internet), and there are geographical and cultural barriers (such as rurality, native communities, diverse population, native languages) that could have limited the collection of information [16,17,18]. Thus, the findings of these studies may not reflect the most recent picture of vaccination in the entire Peruvian territory.

Despite the fact that vaccination coverage against COVID-19 has made great progress in Peru [14], there is still a quarter of the population that is not fully vaccinated. This study aimed to determine the factors associated with complete vaccination in Peruvian adults. The results may help decision makers to design more effective vaccination programs adapted to the local context.

## 2. Materials and Methods

### 2.1. Study Design and Population

This was a cross-sectional study including data extracted from the National Household Survey (ENAHO—acronym in Spanish) 2022, conducted by the National Institute of Statistics and Informatics (INEI—acronym in Spanish) between January and December 2022 [19,20]. The survey was conducted nationwide, covering urban and rural areas in the 24 departments of Peru and in the constitutional province of Callao [19]. The study population included all private households and their occupants residing in urban and rural areas of the country [19]. The participants of the ENAHO 2022 were selected by means of a multistage, stratified, probability sampling, independently performed in each department of Peru [19]. The sampling frame used was based on the information collected in the population and housing censuses. The ENAHO 2022 collected demographic, socioeconomic, and health information through direct interviews with participants [19,20]. The technical report and annual report provided by INEI, which are available online, provide more details on the methodological aspects of the survey [19,20].

The annual sample size of the ENAHO 2022 is 36,822 private households, of which 24,206 correspond to urban areas and 12,616 to rural areas [19]. In the health module of the ENAHO 2022, a total of 113,978 household members were included, of which 85,725 participated in the survey between April and December (in April, questions on vaccination against COVID-19 were added: Did you receive the vaccine against COVID-19? and how many doses did you receive?). Of these, 58,665 participants were aged 18 years or older, excluding missing observations on the COVID-19 vaccination question (*n* = 57), on the number of doses administered (*n* = 9), on educational level (*n* = 78), and on the ethnicity variable (*n* = 50), resulting in a final unweighted sample of 58,471 participants.

### 2.2. Variables and Measurements

#### 2.2.1. Dependent Variable

The dependent variable used in this study was vaccination with 3 or more doses of the COVID-19 vaccine, regardless of which vaccine was given. This variable was coded by assigning a value of 1 to those participants who had received 3 doses or more and a value of 0 otherwise. It is important to note that the COVID-19 vaccination strategy in Peru began in February 2021, following a late procurement of vaccines, and prioritized health personnel and persons aged 60 years or older. In October 2021, the Peruvian Ministry of Health initiated mandatory vaccination with two doses for persons aged 18 years or older nationwide, and in November 2021, the third dose was introduced [13,21]. Subsequently, in 2022, booster doses (third and fourth) were administered due to waves of COVID-19 [22].

#### 2.2.2. Independent Variables

Based on the literature and available data [17,23,24,25,26], a number of individual and contextual variables were included as follows: gender (male, female); age group (18 to 29, 30 to 59, 60 and over); educational level (none or elementary school, primary education, secondary education, higher education); ethnicity (non-native, native); chronic disease (no, yes); physical or psychological limitation (no, yes); type of main occupation (non-health-related activities, health-related activities, no main occupation); poverty status (extreme poverty, non-extreme poverty, non-poor); home without media such as landline, cell phone, TV or internet (no, yes); natural region of residence (jungle, highlands, coast); and area of residence (rural, urban). Furthermore, a variable with the month of the survey (April to December) was created and added in the adjusted analysis to include the variability in vaccination and disease during the year 2022 in the analysis.

### 2.3. Statistical Analysis

We used Stata version 17 software (Stata Corporation, College Station, TX, USA) to download, extract, and analyze the data. The descriptive statistics were calculated, and the findings were presented in tables and figures. Before performing any statistical analysis, we included weights and specified the sample design of the ENAHO 2022 to ensure the representativeness of the results.

The characteristics of the participants were described by the number of participants and the weighted proportion for categorical variables. Furthermore, the proportions of these characteristics were compared with vaccination with three or more doses of the COVID-19 vaccine using the Rao–Scott Chi-square test. The prevalence ratios (PR) and their respective 95% confidence intervals (CI) were calculated using a generalized linear Poisson family model with log link functions to estimate the associations between the independent variables with the dependent variable. All independent variables that were significant in the crude model were entered into the ajusted analysis. A multicollinearity test was performed of each variable, and the results indicated that the variables in the fitted model had a mean of the variance inflation factor (VIF) of 2.45. A VIF score greater than 10 suggests the presence of multicollinearity.

The free software QGIS version 3.30.2 (QGIS Development Team, 2023) was used to plot the distribution of vaccination frequency with three or more doses of the COVID-19 vaccine according to departments. All values of *p* < 0.05 were considered significant.

### 2.4. Ethical Considerations

Approval by an ethics committee was not required, as this study used public and non-identifiable secondary data. The anonymized data are openly available on the INEI microdata website (URL: https://proyectos.inei.gob.pe/microdatos/ (accessed on 12 May 2023)).

## 3. Results

The characteristics of the study population are reported in Table 1. More than half of the population was female (52.6%) and between 30 and 59 years old. Nearly 4 out of 10 individuals had a secondary education (41.3%), and 30.5% had a higher education. Most of the population sample were non-native (72.9%), and more than half had a chronic disease (53.5%). Nearly one-fifth of the population was poor (19.5%), while 3.5% was defined as extremely poor. Most of the individuals lived in an urban setting (81%) and in households without media (97.2%).

**Figure 1:**
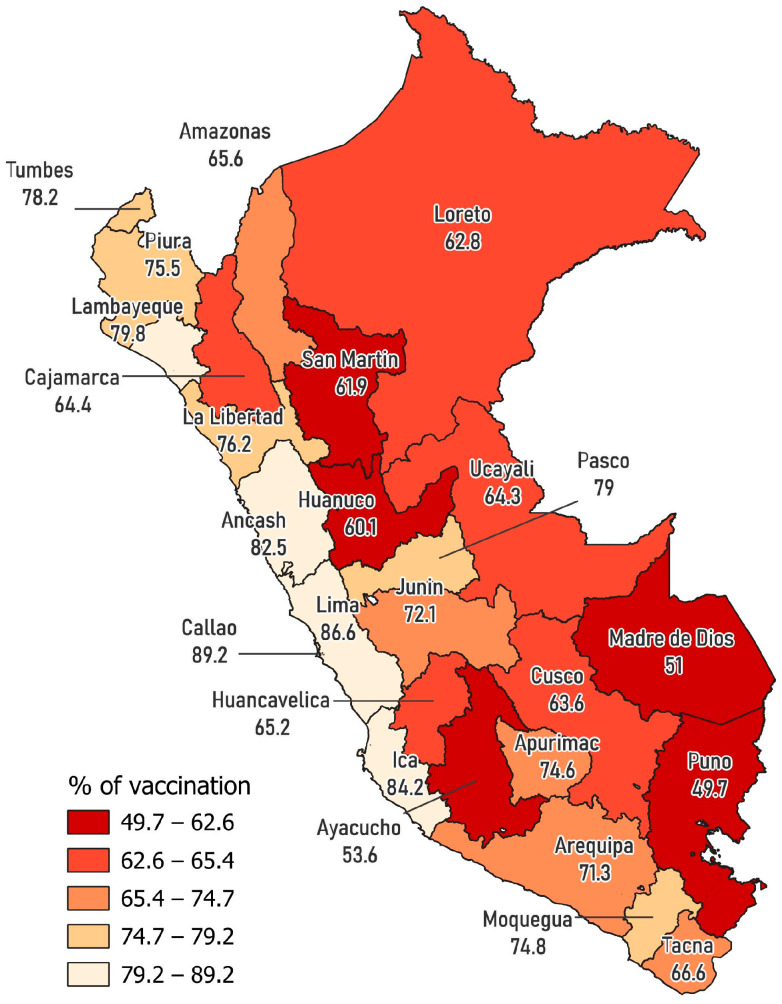
Geographical distribution of vaccination with three doses against COVID-19 by department in Peru: National Household Survey 2022.

Figure 1 shows the geographical distribution of vaccination with three doses against COVID-19 by department in Peru. The departments with the highest frequency of vaccination with three doses were Callao (89.2%), Lima (86.6%), Ica (84.2%), and Ancash (82.5%), while the lowest frequency was reported in the departments of Puno (49.7%), Madre de Dios (51.0%), Huánuco (60.1%), San Martín (61.9%), and Loreto (62.8%).

Table 2 describes the full coverage against COVID-19 according to the socioeconomic characteristics and clinical conditions of the study population. Overall, three quarters of the population received full coverage against COVID-19 (75.8%, 95% CI: 75–76.6%). However, except for the physical or psychological condition, we found differences in the percentage of full coverage according to subgroup populations. For instance, females had a higher percentage of full coverage compared to men (77.1% vs. 74.3%, *p* < 0.001), and older individuals had a higher percentage of full coverage compared to younger individuals (*p* < 0.001). Moreover, individuals with a higher education reported a higher percentage of full coverage compared to individuals with lower educational attainments (*p* < 0.001). When we analyzed ethnicity, by comparing non-native vs. native individuals, we found that non-native individuals had a higher percentage of full coverage (*p* < 0.001). Individuals living in households without media had a higher percentage of full coverage vs. those with media (*p* < 0.001). Individuals classified as not poor reported a higher percentage of full coverage vs. poor or extremely poor individuals (*p* < 0.001). Furthermore, people who lived in urban settings or on the coast reported a higher percentage of full coverage compared with individuals living in the rural settings (*p* < 0.001) or persons living in the jungle or the highlands (*p* < 0.001).

The variables associated with full vaccination coverage are shown in Table 3. In the crude model, all the variables reported a *p*-value less than 0.05, except for physical or psychological limitation, thus, this latter variable was not included in the adjusted model. In the adjusted analysis, we found that individuals 60 years of age or older had a higher probability of being fully covered against COVID-19 compared with individuals aged between 18 and 29 years old (aPR: 1.28, 95% CI: 1.25–1.31). On comparing individuals with a higher education with those with a primary education, the former had a 1.33 times higher probability (95% CI: 1.28–1.39) of being fully covered. Furthermore, non-poor individuals had a 1.21 times higher probability (95% CI: 1.12–1.31) of being fully covered when compared with extremely poor subjects. Finally, persons living in urban settings or on the coast had a greater likelihood of being fully covered compared with those living in rural settings (aPR: 1.11, 95% CI: 1.07–1.14) and when compared with those living in the jungle (aPR: 1.27, 95% CI: 1.27–1.32). In addition, native individuals (aPR: 0.89, 95% CI: 0.87–0.92) and people who lived in households without media (aPR: 0.93, 95% CI: 0.88–0.98) were less likely to be fully covered.

## 4. Discussion

### 4.1. Main Findings

Our findings show that approximately 75% of the Peruvian population has received three or more doses of the COVID-19 vaccine. In addition, some sociodemographic, economic, and geographic factors increase the probability of vaccination in the Peruvian population, while identifying as a native and not having means of communication (internet and television signal) decrease the probability of this outcome. Additionally, the geographical distribution of vaccination indicated that the departments with the highest percentage of complete vaccination were Lima, Callao, Ica, and Ancash, while the lowest percentage was found in departments of the highlands and jungle, such as Puno, Madre de Dios, Huánuco, San Martin, and Loreto.

### 4.2. Comparison with Previous Studies

It was found that 3 out of 4 Peruvians had three or more doses of the COVID-19 vaccine. This finding is higher than that reported by the WHO in regions such as Europe (64.58%), Southeast Asia (68.63%), and Africa (30.55%) [7]. In South America, our finding is lower than that reported by the Pan American Health Organization in countries such as Chile (92.2%), Argentina (83.4%), Uruguay (83.3%), Brazil (79.7%), and Ecuador (79.6%) and higher than that reported in Paraguay (49.1%), Venezuela (49.8%), Bolivia (53.2%), and Colombia (72.2%) [10]. These differences in COVID-19 vaccination coverage among countries could be attributed to problems of delay due to late procurement, affordability, allocation, and production of vaccines in LMIC [27]. However, despite the high vaccine availability and higher prioritization of vaccination policies in high-income countries, low vaccination coverage in these countries could be due to individual factors, such as distrust about vaccines, concern about vaccine adverse events, and that the booster dose is not mandatory, which would limit the uptake of the COVID-19 vaccine [27]. In Peru, our result is similar to that found in the official report of the Ministry of Health, which states that, currently, 74.61% of people have completed the mandatory COVID-19 vaccination schedule (three doses) [14]. In this sense, governmental institutions should redouble their efforts to try to achieve complete vaccination coverage against COVID-19 in the Peruvian territory and ensure that the population is protected against new variants that have negative consequences for public health.

Our study found that there are sociodemographic factors (such as female sex, being 30 years of age or older, having a primary education level or higher), economic factors (having a job as health personnel, and being poor or not poor), personal history (having a chronic disease), and geographic factors (residing in the coast and highlands and in an urban area) that increase the probability of vaccination against COVID-19 in the Peruvian population. These findings are similar to those reported in a systematic review that included 23,000 individuals from 23 countries in various regions of the world, which highlighted that people of male sex, younger, with a lower educational level, and low income were more likely to doubt or not accept vaccination against COVID-19 [28]. Furthermore, our finding that females were more likely to be vaccinated compared to their male counterparts is similar to that reported in individual studies conducted in Canada [26], the United States [25], Ethiopia [24], and Sweden [23]. Although the biomedical literature points out that males possess a higher intention to vaccinate against COVID-19, for the most part, these findings were reported prior to vaccine administration, which could have been modified because intention may be outweighed by vaccination behavior, as described in the literature [29]. Regarding age, younger people were reported to have lower complete vaccination compared to their counterparts. This finding could be since Peru prioritized the administration of vaccines to subpopulations with greater severity and lethality, such as older adults, thus, delaying the administration to younger people [11]. In fact, the literature indicates that COVID-19 severity and mortality increase with age [30]. In addition, older adults have a greater fear of COVID-19, which translates into a greater motivation to be vaccinated [31]. Additionally, people with a higher level of education are more likely to complete the vaccination schedule compared to people with no education. This finding could be explained by a lack of knowledge about the COVID-19 vaccine, leading to higher numbers of low vaccine uptake [32] and the association between education and higher participation in health-promoting behavior, such as vaccination [33].

Likewise, people with chronic diseases showed a higher prevalence of vaccination with three or more doses. This finding could be related to motivation related to reincorporation in their workplaces and greater protection against SARS-CoV-2 virus infection [34], since, as described in several studies [35], this subpopulation has a higher risk of severity and mortality. In relation to economic factors, having a job in the health care setting increased the probability of receiving three or more doses of the COVID-19 vaccine. In Peru, the national vaccination plan against COVID-19 prioritized the administration of the first batch of vaccines to health personnel due to their greater exposure to SARS-CoV-2 infection [11]. This strategy was not only addressed in the Peruvian territory but also by various countries around the world, since the WHO considered this prioritization within the recommendations on vaccination against COVID-19 [4]. On the contrary, the literature points out that health personnel in charge of vaccination have a lower level of acceptance than the general population (65.65% vs. 81.65%), which could generate less confidence in the people who receive the vaccine and lower vaccination rates in the general population [36]. Therefore, strategies that promote vaccination should focus on health personnel who perform not only curative and recovery activities but also preventive strategies such as vaccination against COVID-19. In addition, poor and non-poor people had a higher complete vaccination compared to extremely poor individuals, likely because the Peruvian population with extreme poverty presents greater economic and social vulnerability that limit their access to vaccination [37].

In terms of geographic factors, people residing in the coast and highlands and in an urban area presented higher COVID-19 vaccination rates compared to the jungle region and rural areas. This finding is consistent with the geographical distribution of vaccination, with which departments belonging to the jungle, such as Madre de Dios, Huánuco, San Martín and Loreto, had a lower percentage of complete vaccination. In fact, the official report of the Ministry of Health confirms our findings and indicates that the lowest vaccination coverage is found in the departments of Madre de Dios and Amazonas [14]. Particularly, in the jungle, there are barriers of geographical access, rurality, the presence of ethnic minorities and multiple native languages, and a lack of adequate means of information about vaccines [38,39], which limit optimal vaccination coverage against COVID-19 in the region.

Finally, people who do not have media such as internet and cable television have a lower probability of receiving three or more vaccinations against COVID-19. This could be attributed to the fact that the media are a useful tool to transmit relevant information on health issues (such as vaccination) and promote a healthy lifestyle [40]. In Peru, a communication campaign called “Pongo El Hombro por el Perú. Yo me vacuno” was carried out for a short period of time (23 days) through radio, television, advertising panels, and digital platforms that sought to increase full vaccination coverage throughout the Peruvian territory [41]. In fact, after the communication campaign (in the last days of June), there was an increase in the doses administered of the vaccine against COVID-19 [14]. In this sense, communication campaigns should be permanently transmitted and in a way that is accessible to people who do not have means of communication, such as television or internet, to improve coverage in the population that is still not fully vaccinated.

### 4.3. Public Health Implications

The national vaccination plan against COVID-19 has achieved great progress in the Peruvian territory. However, our results highlight the need to continue with this strategy to achieve complete vaccination coverage in the entire population. Within the WHO recommendations on vaccination against COVID-19, it is postulated that countries should maintain and improve the drive to achieve coverage of all age groups [4]. This recommendation should be embraced by Peruvian governmental institutions to maintain vaccination campaigns, especially in regions with low complete vaccination coverage. In the Peruvian jungle, in particular, mass vaccination campaigns should be reincorporated and restructured considering the main constraints (access, information, rurality, and native communities) observed in this region in order to achieve optimal coverage throughout the territory. Finally, vaccination communication campaigns should be reincorporated in various regions and include culturally inclusive strategies (in Quechua or Aymara language and respecting their beliefs) to improve acceptance of the COVID-19 vaccine.

### 4.4. Strengths and Limitations

One of the main strengths of our study is the use of a nationally representative Peruvian survey that allowed us to provide a current overview of the progress of vaccination in the Peruvian territory. In addition, our study is one of the first investigations that incorporates population-based data and direct interviews from one of the most important surveys in Peru. However, our study is not without limitations. First, the data were collected based on self-reports of the interviewees. This is often prone to recall and social desirability bias. Second, the cross-sectional nature of the study does not allow causal inferences to be made. Finally, there are individual variables, such as self-perception of health, fear about the vaccine, and a history of COVID-19, among others, which were not available or with a very low number of observations in the ENAHO.

## 5. Conclusions

Our findings indicate that vaccination of three or more doses against COVID-19 in Peru reached a coverage of 75%. Based on the calculated aPR, sociodemographic (such as female sex, being 30 years of age or older, having a primary education level or higher), economic (having a job as health personnel, and being poor or not poor), individual (having a chronic disease), and geographical (residing in the coast and highlands and in an urban area) factors that increased the probability of obtaining full vaccination in our study should be considered for the formulation of strategies to achieve full vaccination coverage. Likewise, our spatial analysis reported that the lowest vaccination rates were found in the departments belonging to the jungle region, suggesting the need to restructure the current health strategies aimed at improving geographic access and information systems and promoting interculturality in vaccination campaigns.

## Figures and Tables

**Table 1:** Distribution of the geographic and socioeconomic characteristics of the adults surveyed in the National Household Survey 2022.

Characteristics	*n* (*n* = 58,471)	%
Gender		
Male	27,782	47.4
Female	30,689	52.6
Age group (years)		
18–29	13,792	24.6
30–59	30,964	52.8
60 or more	13,715	22.5
Educational level		
None or elementary school	3429	5.2
Primary education	15,314	23.0
Secondary education	22,148	41.3
Higher education	17,580	30.5
Ethnicity		
Non-native	40,903	72.9
Native	17,568	27.1
Chronic disease		
No	26,820	46.5
Yes	31,651	53.5
Physical or psychological limitation		
No	54,727	94.2
Yes	3744	5.8
Type of main occupation		
Non-health-related activities	44,696	73.8
Health-related activities	1026	1.7
No main occupation	12,749	24.5
Poverty status		
Extreme poverty	2606	3.9
Non-extreme poverty	9927	19.5
Non-poor	45,938	76.6
Home without media		
No	56,112	97.2
Yes	2359	2.8
Natural region of residence		
Jungle	12,199	11.3
Highlands	20,058	30.7
Coast	26,214	58.0
Area of residence		
Rural	19,054	19.0
Urban	39,417	81.0

The analysis only included Peruvians aged 18 years and over. *n*: number of unweighted observations. %: Row percentage weighted by expansion factor and sample specifications of National Household Survey 2022. % were rounded to 1 decimal places.

**Table 2:** The frequency of individuals unvaccinated and vaccinated with three doses according to geographic and socioeconomic characteristics: National Household Survey 2022.

Characteristics	Three or More Doses of Vaccine Against COVID-19		
	No (*n* = 15,721)	Yes (*n* = 42,750)	
	% (95% CI)	% (95% CI)	*p*-Value *
Overall	24.2 (23.4–25.0)	75.8 (75.0–76.6)	
Gender			
Male	25.7 (24.7–26.6)	74.3 (73.4–75.3)	<0.001
Female	22.9 (22.0–23.8)	77.1 (76.2–78.0)	
Age group (years)			
18–29	29.1 (27.7–30.5)	70.9 (69.5–72.3)	<0.001
30–59	25.0 (24.1–25.9)	75.0 (74.1–75.9)	
60 or more	17.1 (16.0–18.2)	82.9 (81.8–84.0)	
Educational level			
None or elementary school	36.5 (34.2–39.0)	63.5 (61.0–65.8)	<0.001
Primary education	32.9 (31.5–34.3)	67.1 (65.7–68.5)	
Secondary education	26.2 (25.1–27.3)	73.8 (72.7–74.9)	
Higher education	12.9 (12.0–13.8)	87.1 (86.2–88.0)	
Ethnicity			
Non-native	20.1 (19.3–21.0)	79.9 (79.0–80.7)	<0.001
Native	35.2 (33.7–36.8)	64.8 (63.2–66.3)	
Chronic disease			
No	27.7 (26.7–28.8)	72.3 (71.2–73.3)	<0.001
Yes	21.2 (20.3–22.1)	78.8 (77.9–79.7)	
Physical or psychological limitation			
No	24.1 (23.3–25.0)	75.9 (75.0–76.7)	0.202
Yes	25.5 (23.4–27.7)	74.5 (72.3–76.6)	
Type of main occupation			
Non-health-related activities	26.1 (25.2–27.1)	73.9 (72.9–74.8)	<0.001
Health-related activities	8.0 (5.8–10.9)	92.0 (89.1–94.2)	
No main occupation	19.5 (18.3–20.7)	80.5 (79.3–81.7)	
Poverty status			
Extreme poverty	46.3 (42.1–50.5)	53.7 (49.5–57.9)	<0.001
Non-extreme poverty	32.4 (30.6–34.3)	67.6 (65.7–69.4)	
Non-poor	21.0 (20.2–21.8)	79.0 (78.2–79.8)	
Home without media			
No	23.7 (22.9–24.6)	76.3 (75.4–77.1)	<0.001
Yes	40.8 (37.5–44.3)	59.2 (55.7–62.5)	
Natural region of residence			
Jungle	40.1 (38.1–42.2)	59.9 (57.8–61.9)	<0.001
Highlands	33.8 (32.2–35.4)	66.2 (64.6–67.8)	
Coast	16.0 (15.1–17.0)	84.0 (83.0–84.9)	
Area of residence			<0.001
Rural	40.9 (39.3–42.6)	59.1 (57.4–60.7)	
Urban	20.3 (19.4–21.2)	79.7 (78.8–80.6)	

The analysis only included Peruvians aged 18 years and over. *n*: number of unweighted observations. %: Row percentage weighted by expansion factor and sample specifications of the National Household Survey 2022. % were rounded to 1 decimal places. * *p* Values were based on Rao–Scott Chi-square tests for complex survey data. Statistically significant: *p* < 0.05.

**Table 3:** Crude and adjusted analysis of factors associated with COVID-19 vaccination: National Household Survey 2022.

Variable	Crude Model		Adjusted Model	
	PR (95% CI)	*p*-Value	aPR (95% CI)	*p*-Value
Gender				
Male	Reference		Reference	
Female	1.04 (1.03–1.05)	<0.001	1.04 (1.03–1.06)	<0.001
Age group (years)				
18–29	Reference		Reference	
30–59	1.06 (1.04–1.08)	<0.001	1.10 (1.08–1.12)	<0.001
60 or more	1.17 (1.15–1.19)	<0.001	1.28 (1.25–1.31)	<0.001
Educational level				
None or elementary school	Reference		Reference	
Primary education	1.06 (1.02–1.10)	0.004	1.07 (1.03–1.11)	0.001
Secondary education	1.16 (1.12–1.21)	<0.001	1.16 (1.12–1.21)	<0.001
Higher education	1.37 (1.32–1.43)	<0.001	1.33 (1.28–1.39)	<0.001
Ethnicity				
Non-native	Reference		Reference	
Native	0.81 (0.79–0.83)	<0.001	0.89 (0.87–0.92)	<0.001
Chronic disease				
No	Reference		Reference	
Yes	1.09 (1.07–1.11)	<0.001	1.04 (1.03–1.06)	<0.001
Physical or psychological limitation				
No	Reference		Not included	
Yes	0.98 (0.95–1.01)	0.213		
Type of main occupation				
Non-health-related activities	Reference		Reference	
Health-related activities	1.25 (1.21–1.28)	<0.001	1.05 (1.02–1.08)	0.001
No main occupation	1.09 (1.07–1.11)	<0.001	1.00 (0.99–1.02)	0.868
Poverty status				
Extreme poverty	Reference		Reference	
Non-extreme poverty	1.26 (1.16–1.36)	<0.001	1.12 (1.04–1.20)	0.001
Non-poor	1.47 (1.36–1.59)	<0.001	1.21 (1.13–1.30)	<0.001
Home without media				
No	Reference		Reference	
Yes	0.78 (0.73–0.82)	<0.001	0.93 (0.88–0.98)	0.006
Natural region of residence				
Jungle	Reference		Reference	
Highlands	1.11 (1.06–1.15)	<0.001	1.14 (1.10–1.18)	<0.001
Coast	1.40 (1.35–1.45)	<0.001	1.27 (1.23–1.32)	<0.001
Area of residence				
Rural	Reference		Reference	
Urban	1.35 (1.31–1.39)	<0.001	1.11 (1.07–1.14)	<0.001

Weighting factors and sample specifications of ENAHO were included for all estimates. ENAHO: Encuesta Nacional de Hogares. PR: prevalence ratio. aPR: adjusted prevalence ratio. CI: confidence interval. Adjusted models were made for all variates with *p*-values less than 0.05, and the month of the survey was included as an adjustment variable. Estimates were rounded to 2 decimal places.

## Data Availability

The ENAHO 2022 microdata were obtained from the INEI website (http://iinei.inei.gob.pe/microdatos/ (accessed on 12 May 2023)). The ENAHO 2022 databases can be obtained by accessing the survey query tab and selecting the “ENAHO Metodología ACTUALIZADA” survey, “Condiciones de Vida y Pobreza—ENAHO, and year “2022”.
